# Perinatal biology symposium: perinatal programming—challenges and solutions for optimizing long-term health

**DOI:** 10.1093/jas/skag328

**Published:** 2026-10-08

**Authors:** Ryan L Ashley, Dustin T Yates

**Affiliations:** Department of Animal and Range Sciences, New Mexico State University, Las Cruces, NM 88003, United States; Department of Animal Science, University of Nebraska-Lincoln, Lincoln, NE 68583, United States

**Keywords:** developmental origins of health and disease (DOHaD), gamete heath, intrauterine growth restriction, low birthweight, maternofetal medicine, placental development

## Abstract

Perinatal biology encompasses the coordinated sequence of parental, placental, fetal, and neonatal processes that collectively determine reproductive success and form the basis for lifelong health of the offspring. Recent advances in molecular biology, reproductive technologies, and systems biology have transformed our understanding of how events occurring before conception and throughout gestation influence pregnancy outcomes across mammalian species. The invited reviews that comprise this Perinatal Biology collection highlight emerging concepts in assisted reproductive technologies, maternal endocrine patterns, placental biology, infectious disease, and gamete physiology. Collectively, these articles demonstrate how successful pregnancy depends upon dynamic communication among maternal and paternal, placental, and fetal compartments, as none of these functions independently. Across species, common biological themes emerge around mitochondrial function and metabolic adaptability, immune and endocrine regulation, angiogenesis, and cellular plasticity. The collection also emphasizes the importance of comparative animal models, multi-omics technologies, and integrative physiology for identifying conserved mechanisms that underlie pregnancy outcomes. Together, this collection illustrates the expanding scope of perinatal biology and establishes a framework for translational One-Health research that benefits human medicine and animal agriculture.

## Teaser

The Perinatal Biology Symposium is a triennial international meeting of scientists and clinicians working to improve perinatal outcomes in humans and animals. This collection is a representation of the cutting-edge advancements in knowledge that continue to accelerate this unique and critical field.

## Introduction

This collection was inspired by the expertise and knowledge shared at the 2025 Perinatal Biology Symposium (see Symposium Abstracts, *J. Anim. Sci.* vol. 104 suppl. 1, 2026), a triennial scientific gathering created to grow and strengthen connections among perinatal researchers around the globe and to communicate new advances in perinatal science. The initial meetings date back to 2000 and were developed as FASEB summer conferences. However, FASEB conferences did not prioritize promoting the work of early career investigators or trainees and focused exclusively on biomedical perspectives. For these and other reasons, the conference separated from FASEB after the 2004 meeting, and the 1^st^ stand-alone Perinatal Biology Symposium was held in 2007 in Aspen, CO. The meetings have been held in the Aspen/Snowmass area every 3^rd^ year since.

Perinatal biology is a preeminent example of One-Health science, and the Symposium leverages this approach by highlighting broad areas of intersection among human, animal, and environmental health factors. As one example, outcomes of environment-influenced fetal metabolic programming are strikingly similar between sheep/cattle exposed to seasonal heat and women from regions where socioeconomic status requires that they continue in laborious jobs throughout gestation. As another example, early nutritional needs are similar in preterm human infants and preterm piglets. For humans and animals alike, potentially harmful environmental elements as obvious as climate and as inconspicuous as chemicals in car seat foam continue to threaten perinatal health.

## Perinatal programming and long-term health

The theme of this collection and the 2025 Symposium it is based on are derived from epidemiological studies that show clear associations between environment-altered fetal/early neonatal development and subsequent risk for health disorders (Hales and Barker 1992; Barker et al. 1993). Consequences of these programmed pathologies worsen as offspring age and ultimately manifest in conditions like diabetes, obesity, immune dysfunction, behavioral and cognitive disorders, subfertility, and cardiovascular disease, to name but a few. In humans, such conditions shorten lifespan and reduce quality of life. In livestock, the same underlying biomechanisms increase early morbidity and mortality, reduce growth efficiency, and diminish the volume, quality, and value of food produced from each animal (De Blasio et al. 2007; Bell and Greenwood 2016; Greenwood and Bell 2019). Prenatal and early postnatal development in mammals depends on intricate interactions among maternal, placental, and fetal systems. The expansive and complex nature of this network makes it generally necessary to study each component individually. However, periodic opportunities to consider a holistically integrated view of the perinatal paradigm are important to maintain the effectiveness of research efforts that focus on discrete components and outcomes. The Perinatal Biology Symposium is widely recognized as a productive mechanism for facilitating such integration of scientific thought across the fundamental areas summarized in Table 1. The invited speakers that contributed to this collection represent broad expertise in the cellular mechanisms for normal and pathological maternal, placental, fetal, and neonatal outcomes. Their work is integral to understanding how influences on intrauterine and early postnatal environments affect these outcomes in newborn infants, livestock, animal research models, and even wildlife.

**Table 1:** Strategic integration of fundamental areas of perinatal biology.

Integrated area	Synopsis
**Biomedical & agricultural biosciences**	Developmental mechanisms affecting long-term health and welfare are common among humans and other mammalian species. Scientific discoveries in perinatal health are likewise relevant to both.
**Clinical, basic, & applied research**	Understanding complex perinatal events and the conditions affecting them requires strong interdisciplinary networks of clinicians and scientists from myriad disciplines across agricultural, biomedical, and environmental fields.
**Medical centers, land-grant universities, & other research institutions**	Research institutions differ broadly in capacity and resources. Scientific meetings like the Perinatal Biology Symposium provide opportunities for scientists at different institution types to connect and synergize capabilities to increase impact.
**Leading experts, early-career investigators, & trainees**	Sustaining impactful perinatal research, education, and knowledge requires regular opportunities for established researchers, early-career scientists, and trainees to interact and learn from each other.

## Collection topics

Core physiological mechanisms underlying maternal, fetal, and postnatal health outcomes are well-conserved among mammalian species. As such, the basic biological factors that determine lifelong wellness, metabolic function, growth, body composition, and disease predispositions are comparable among humans and animals (Reynolds et al. 2010; Beede et al. 2019). The broad relevance integrated into this collection illustrates the power of effective transdisciplinary scientific interaction and its benefit on perinatal outcomes. The following major areas are covered by the authors.

### Pregnancy as an integrated biological system

Perhaps the strongest theme emerging from this collection is a recognition that healthy pregnancies cannot be characterized by phenomena occurring within a solitary organ or tissue or individual window of time. Rather, successful progression of pregnancy requires synchronized regulation of endocrine regulation from maternal, placental, and conceptus origins. These papers capture how critical events such as placentation and fetal organogenesis require complex coordination of intricate endocrine patterns, tissue morphogenesis and hypertrophy, and regional nutrient supply. They also detail how such events are facilitated by properly timed changes in factors such as immune tolerance, angiogenesis, and tissue-specific metabolic flex. This network of regulatory input arises from dynamic participation of tissues as disparate as the maternal pancreas, the myriad placental cell types, the developing fetal endocrine tissues, and even paternal gametes, all of which provide essential, unique influence to regulate developmental trajectories. The overarching thread of this collection is its emphasis on comparative reproductive biology. It collectively incorporates evidence from humans, mice, sheep, pigs, horses, cattle, guinea pigs, nonhuman primates, and even polar bears to connect an understanding of pregnancy events. This integration reflects the increasingly interdisciplinary nature of perinatal biology.

### Influence of parental factors & status

Ensuring appropriate physiological changes and adjustments by the mother’s body before and during pregnancy is a critical initial consideration for perinatal success. Indeed, maintaining a healthy status amid marked changes necessitated by pregnancy is perhaps the ultimate foundation for successful reproductive outcomes. Investigations to this end take many forms. The review by Pszczolkowski et al. (2026) from the lab of Emilyn Alejandro at the University of Minnesota exemplifies this systematic perspective by reframing gestational diabetes mellitus as failed maternal pancreatic β cell adaptation to the pregnancy state rather than just maternal insulin resistance. Pregnancy requires remarkable expansion of β cell mass and insulin secretion capacity in parallel with inevitable placenta-induced insulin resistance. Discoordination of these events results in gestational diabetes and poor maternal-fetal nutrient exchange. Maternal endocrine flexibility does not just apply to humans, as illustrated by the contribution from Wojtusik et al. (2026) from Omaha’s Henry Doorly Zoo & Aquarium. In this article, the authors characterize the necessary coordination of major reproductive and trophic hormones in polar bears that permit successful establishment and progression of pregnancy. Endocrine regulation of pregnancy is quite interdependent on maternal nutrition, and both are reflected in perinatal outcomes. The Gomez-Sharma and McCurdy (2026) review from the lab of Carrie McCurdy at the University of Oregon describes the impact of maternal nutritional imbalance caused by high-caloric diets on fetal mitochondrial development. They detail literature across rodents, livestock, and primates implicating western-style diets in persistent disruptions in mitochondrial abundance, architecture, and functional capacity within fetal skeletal and cardiac muscle. The paper from the Caitlin Cadaret lab at Colorado State University (Stucke et al. 2026) further describes the long-term consequence of maternal malnutrition by extending the observations to poor postnatal lean tissue growth and diminished thermoregulation capacity.

While much perinatal biology focuses on maternal and fetal interactions, two reviews broaden pre-conception considerations to include the under-investigated interactions between paternal and fetal outcomes. The review from the lab of Maria Gracia Gervasi at the University of Connecticut (Arroyo-Salvo et al. 2026) describes Sperm Energy Restriction and Recovery (i.e., SER), a process whereby transient metabolic conditioning of sperm via brief nutrient restriction improves developmental competence of the conceptus following *in vitro* fertilization. Conversely, the comprehensive review of porcine Leydig cell biology by Amy Desaulniers at the University of Nebraska-Lincoln (Desaulniers et al. 2026) illustrates how fetal endocrine programming impacts neonatal Leydig development and, in turn, puberty and adulthood fertility of the male. These manuscripts illustrate that paternal conditions influence perinatal outcomes but also that perinatal conditions influence the paternal potential of the offspring.

### The central role of the placenta

This collection characterizes the placenta’s role as the integrator of maternal and fetal factors and influences. In addition to nutrient and gas transport, the placenta fulfills dynamic endocrine, metabolic, and immunological functions that guide fetal growth and developmental programming (Ashley et al. 2023). Manuscripts within this collection highlight convergent mechanisms regulating placental function across species, including growth factor signaling, extracellular matrix remodeling, cellular differentiation, immune regulation, oxidative stress, and mitochondrial function, which emerge as recurring features of placental dysfunction. The review from Lisa Vrooman’s lab at Oregon Health Science University (Hickey et al. 2026) examining placental outcomes of assisted reproductive technologies describes remarkably conserved mechanistic regulation of placental metabolism and vascular development among different species and clinical approaches. Clyde Wright’s lab at the University of Colorado Anschutz Medical Campus introduces for consideration the potential pharmaceutical toxicity of the placenta due to acetaminophen use during pregnancy (Etuka et al. 2026). Rather than considering the placenta solely as a passive barrier, this review highlights placental CYP2E1-mediated metabolism, trophoblast dysfunction, and aberrant angiogenesis as potential mechanisms linking maternal acetaminophen use with adverse pregnancy outcomes. The review of Zika virus infection by Sathish Kumar Natarajan’s lab at the University of Nebraska-Lincoln (Krishnamoorthy and Natarajan 2026) demonstrates how this infectious disease disrupts many of these same biological pathways within the placenta, which yields excessive trophoblast apoptosis, inflammation, and vascular dysfunction. These insults impair placental integrity and facilitate fetal infection by the virus. These reviews show that diverse environmental insults often converge on common placental mechanisms. However, biological responses to pregnancy complications are not always uniform. The review examining sex-specific placental adaptations by Helen Jones’ lab at the University of Florida (Casarotto et al. 2026) demonstrates that fetal sex fundamentally influences placental responses to stress. For example, the female placenta exhibits greater plasticity in angiogenesis, growth factor sensitivity, and metabolic regulation, whereas the male placenta generally prioritizes fetal growth even at the expense of resilience to placental insult.

### Emerging treatments & technologies

The collection emphasizes that technological advances are fundamentally changing research tactics and clinical approaches throughout the field. Transcriptomics, proteomics, single-cell sequencing, spatial transcriptomics, metabolomics, multiplex endocrine profiling, and advanced reproductive technologies now permit investigation of pregnancy with unprecedented molecular resolution. These approaches reveal previously unrecognized interactions among regulatory and functional systems and provide new opportunities for precision interventions in humans and animals. For example, Hickey et al. (2026) and Arroyo-Salvo et al. (2026) describe how assisted reproductive technologies influence placental development from the earliest stages of embryogenesis. They highlight studies that have used transcriptomic and proteomic techniques to identify complex changes in placental anatomy and function from common perturbations across mammalian species, which provides new insight into the lasting consequences of placental and fetal development. Etuka et al. (2026) describe evolving dogma regarding the use of common over-the-counter medications during pregnancy based on their potential impact on the unique tissues of the placenta, which could have far-reaching implications on global maternofetal health care.

## Final thoughts and future perspectives

Perinatal biology has evolved from a narrow initial focus on fetal growth, birthweight, and early neonatal survival into an integrative field capable of examining how parental physiology, placental development, fetal adaptations, and early postnatal life interact to shape lifelong health outcomes. Advances in omics and other technologies have revealed that pregnancy represents a dynamic biological continuum extending from gamete maturation through the early neonatal period. Historically, reproductive disorders were investigated independently, but increasing evidence now establishes that these conditions are interconnected by common underlying molecular mechanisms. Understanding these communal mechanisms has become a key focus for research approaches in biomedicine and food animal production. The reviews assembled in this Perinatal Biology collection highlight key emerging concepts and provide complementary perspectives spanning the diverse subjects of perinatal success. Collectively, they illustrate the remarkable evolution of reproductive biology from descriptive physiology toward integrated systems biology and show the power of integrating comparative animal models with emerging multi-omics technologies to identify conserved mechanisms regulating reproductive success. The research streams described in this collection also provide reason for optimism regarding continued improvement of perinatal health via highly effective animal models, comprehensive omics approaches, and bold approaches toward common environmental limitations to early health.

## Abbreviations

CYP2E1: Cytochrome P450 2E1
FASEB: Federation of American Societies for Experimental Biology
SER: sperm energy restriction and recovery
